# Reply to: ‘comments on ‘effects of refractive accommodation on subfoveal choroidal thickness in silicone oil-filled eyes”

**DOI:** 10.1186/s12886-022-02686-3

**Published:** 2022-12-08

**Authors:** Ying Yan, Bo Zeng, Chengyuan Gao, Yanping Song, Xiao Chen

**Affiliations:** 1 Department of Ophthalmology, PLA Middle Theater General Hospital, 627 Wuluo Road, Wuhan, 430070 China; 2 Southern Medical University, Guangzhou, China

**Keywords:** Choroidal mechanism of accommodation, Silicone oil, Refractive accommodation

## Abstract

In this article, we answered the questions in Lei Gao et al.’s comments on the "effects of refractive accommodation on subfoveal choroidal thickness in silicone oil-filled eyes" one by one.

We appreciate Lei Gao et al.’s comments on our study regarding the effects of refractive accommodation on subfoveal choroidal thickness (SFCT) in silicone oil (SO)-filled eyes.

We realise that accommodation partly depends on the change in lens refractive power according to the Helmholtz theory of accommodation [1, 2]. Accommodation is associated with many theories, including choroidal mechanism [3], ocular elongation and the scleral growth mechanism [4], and the classic Helmholtz [5] theory. Wallman pointed out that choroidal changes were transitory and rapid phenomena that compensated for myopic and hyperopic defocus [6]. For example, according to a study [7] performed on chickens, rapid choroidal thickening was produced at an average rate of 53 ± 36 μm/day after the positive lenses were removed. In our study, the mean SFCT in the SO-filled eyes increased within 24 h from 221.52 ± 38.41 μm to 269.28 ± 36.90 μm after refractive error (RE) correction. The rapid choroidal mechanism of accommodation complements other slower recovery mechanisms, such as ocular elongation [6].

The lack of long-term follow-up on the changes in SFCT after the correction of RE in the SO-filled eyes was due to considerations of compliance and the safety of our patients. We note from Lei Gao’s literature reports [8] that the change in lens refractive power in a short time mainly relies on the change of the lens’s shape. However, the difference in lens thickness before and after wearing contact lenses was not the topic of our study. We focused on the effects of refractive accommodation on SFCT in SO-filled eyes because the choroid is a vascular layer that supplies oxygen and nutrients to the outer part of the retina. Previous studies have shown that the reduction in SFCT of SO-filled eyes after pars plana vitrectomy (PPV) might be related to the negative effects of intravitreal SO. However, the possible mechanism of SO tamponade leading to a decrease in choroid thickness is not clearly understood. Based on our study, we infer that the reduction in the SFCT of SO-filled eyes may primarily be due to the hyperopia caused by SO, which can be reversed after RE correction.

Regarding the diurnal variation in choroidal thickness (CT), all the examinations were performed between 9 a.m. and 10 a.m.

To avoid the accommodation of the SO-filled eyes, taking the healthy eye as the reference, the healthy eye was covered, while the SO-filled eyes wore soft contact positive lenses. Furthermore, the other eye was covered while the subject eye was examined by optical coherence tomography. This was not described in details in the methods section of the original article.

It should also be pointed out that 4.5 D was not the amplitude of accommodation. It was the RE of the eyes after the SO tamponade screened with an autorefractor (Canon Autorefractor RK-F1, Canon Inc. Ltd., Tochigiken, Japan). The mean RE of the SO-filled eyes was + 6.64 ± 1.25 D, ranging from + 4.5 to + 9.5 D (Table 1 in the original article).

## Data Availability

The datasets used and/or analyzed during the current study are not publicly available due to their containing information that could compromise the privacy of research participants, but are available from the corresponding author Xiao Chen (cxfn817@163.com) in a de-identified manner on reasonable request.

## Abbreviations

CT: Choroidal thickness
PPV: Pars plana vitrectomy
RE: Refractive error
SFCT: Subfoveal choroidal thickness
SO: Silicone oil
